# Reduced Cancer Screening Due to Lockdowns of the COVID-19 Pandemic: Reviewing Impacts and Ways to Counteract the Impacts

**DOI:** 10.3389/fonc.2022.955377

**Published:** 2022-07-29

**Authors:** Tuan Luu

**Affiliations:** Management & Marketing Department, Swinburne University of Technology, Melbourne, VIC, Australia

**Keywords:** cancer screening, cancer diagnosis, COVID-19 pandemic, prognosis, mortality

## Abstract

The COVID-19 pandemic has created disruptions in health services in general and cancer screening and diagnostic services in particular, leading to diminished cancer screening participation rates. This paper aims to seek insights into impacts that the pandemic has had on cancer screening, impacts that reduced cancer screening may have in the long run, and how to address such impacts. The paper demonstrates that reduced cancer screening in the pandemic is likely to result in enhanced demands for cancer screening in the new normal, enhanced demands for resources to address such demands, and poor prognosis due to stage migration of cancer diseases. Some measures are recommended for counteracting these impacts.

## Introduction

December 2019 witnessed the emergence of coronavirus 2019 disease (COVID-19). The outbreak of this disease was officially announced as a pandemic by the World Health Organization (WHO) on 11 March 2020 (1). Prior to the availability of vaccines and therapies, the mainstream reaction to the pandemic has been social distancing and quarantine due to high contagion of this virus (2). Such measures taken to control the virus spread have influenced almost everyone (3). Lockdowns and stay-at-home orders have been implemented in numerous places in any country when the case number has increased across it (4). In addition to temporary closure of public facilities and social events, health services have been greatly impacted by the pandemic (5).

Guidance has been issued by Disease Control and Prevention centers to guide people to implement exposure mitigation (6). Older adults and high-risk individuals for severe COVID-19 complications such as individuals with comorbidities have been given special attention by health services (7). Accordingly, non-emergency clinical appointments in clinics and hospitals have been substantially minimized (7). These changes have directly and negatively affected patients in general, cancer patients in particular, as well as those with demands for cancer screening especially due to suspicious symptoms (7–9). Indeed, during the progress of the pandemic, a substantial reduction has been reported for rates of cancer screening testing and diagnoses of precancerous and cancerous lesions for both new and recurring incidence (3, 10). This essay aims to obtain insights into the impacts that the pandemic has exerted on cancer screening and diagnosis, what impacts this will likely have in the long run, and how to address these impacts.

## Reasons for Reduced Cancer Screening

Reduction in cancer screening and diagnosis during the pandemic can be attributable to several reasons. First, compared to pre-pandemic, promotion activities for cancer screening through mass media as well as from hospitals have been less active (11). Second, cancer-related patient encounters, which have been substantially diminished during the pandemic, have contributed to mitigated cancer screening and diagnosis (10). Reduced patient encounters have resulted from their hesitation to visit outpatient services by virtue of apprehension related to contracting or transmitting coronavirus (12). This anxiety prevents patients from seeking care not only for routine issues such as follow-up screening but likewise for emergent issues such as suspicious cancer symptoms (13). Such a concern is further exacerbated when high-risk people for cancer or those with comorbidities such as cancer have been advised to self-isolate and minimize social contacts due to their high risk of contracting coronavirus and developing severe disease (14–16).

Third, decreased patient encounters have stemmed from restrictions on visits to hospitals or outpatient clinics during lockdowns (11, 17). Patient encounters are more inclined to be disrupted or delayed particularly for potential cancer patients who need initial diagnosis, cancer screening, or therapy initiation, albeit scheduled visits for cancer therapies or follow-up tend to be continued for patients with established diagnoses for cancer (10). During the pandemic, numerous healthcare providers have postponed the supply of many crucial cancer screening tests such as colonoscopy and mammography (10), which might have led to delayed cancer diagnoses and surgical treatments (3).

Last, in a number of Western nations where healthcare is largely tax-based and public, general practitioners serve as gatekeepers, through which access to secondary care can be made. Regardless of the utility of primary healthcare system, in-person consultations for primary cancer care are restricted during lockdowns of the pandemic (16). This is a barrier to people who would like to obtain non-acute consultations from the primary cancer care for mild but suspicious symptoms (16).

## COVID-19 Impacts on Cancer Screening and Diagnosis

In the U.S., screening for lung, prostate, colon, and breast cancer were found to diminish by 56%, 74%, 75%, and 85% respectively at the pandemic peak in April 2020 (12). Cancer screenings that utilize blood tests such as for prostate cancer (testing for prostate-specific antigen) have tended to have a lower deficit compared to cancer screenings that involve procedures such as colonoscopy and mammography (4). Particularly, diagnostic mammograms and screening mammograms demonstrated drops in number by 38% and 58% 20 weeks after 11 March 2020 in the U.S (9).

Since temporary suspension of cancer screening programs in March 2020, cancer screening invitations have not been distributed to around 3 million people in the UK compared to around 210,000 people participating in screening programs for cervical, breast, and bowel cancer prior to the pandemic (18). The worst hit was endoscopy services with the decrease in the number of endoscopies for bowels by 90% in April 2020 (19).

Disruptions in primary cancer screening were implemented in Canada and the Netherlands from March to May 2020 (20). Specifically, in the Netherlands, roughly 65% drop occurred in screening through colonoscopy, and a sharp drop in diagnosis of colorectal cancer among 55–75 year-old people occurred from March to June 2020 (21). In Australia, restrictions on the healthcare system influenced numerous diagnostic follow-up services, albeit primary cancer screening has been less affected during the pandemic outbreak (20). For instance, a drop by 55% occurred to the number of diagnostic colonoscopies from March to April 2020 in Australia (20).

## Long-Term Impacts of Reduced Cancer Screening

Reduced screening and diagnoses for cancer during the pandemic may exert some long-term impacts on patients as well as healthcare systems. First, it is a likelihood of enhanced demands for cancer screening tests and cancer diagnostic investigations, which have been rescheduled, delayed, or cancelled during the pandemic (10). When diagnostic procedures become available due to easing of pandemic restrictions, increased resources, including human resources and laboratory capacity, have a propensity to take place to respond to augmented demands for cancer screening as well as therapies (10).

Second, delayed or cancelled cancer screening, particularly through precision prevention technologies at diagnostic centers, during the pandemic might continue to influence high-risk individuals even when lockdowns were eased. These individuals were inclined to be no-shows for cancer screening through such technologies. Research demonstrated that high-risk individuals were less likely to approach low-dose CT (LDCT) screening compared to pre-pandemic, regardless of the utility of screening through LDCT in identifying RADS 4 nodules in lungs, enhancing referrals for intervention, and mitigating mortality of lung cancer by around 20% (7).

Third, the impact of the pandemic could be observed on the prognoses of cancer patients across types of cancer on account of delayed or disrupted cancer screenings or diagnoses (10). Such disrupted cancer screenings will likely leverage a migration of cancer diseases to later stages as well as cancer mortality in the years following the COVID-19 outbreak (12). Disrupted cancer screenings with delayed cancer diagnoses and treatments as natural consequences would lead to more complicated management and care for later-stage cancer with reduced possibility of patients’ responsiveness to therapies and survival (12).

Studies have provided some observations for potential links between disrupted or reduced cancer screenings, diagnoses, or treatments and mortality rates. For instance, a work of de Jonge et al. (20) demonstrated that disrupted screening services for colorectal cancer up to twelve months could lead to 1360–1762 excess deaths in the Netherlands, 2366 in Canada, and 3968 in Australia. In the period after the disruptions, hundreds of extra deaths were estimated to occur as a result of decreased cancer screening participation (20). Degeling and colleagues’ (22) study in Australia also reported that 90 excess deaths would occur in the following five years if diagnoses and treatments were delayed three months for lung, colorectal, breast, and melanoma cancer identified in 2020, and 350 excess deaths would occur for six-month delays for such cancer types. Hanna’s (23) study in the UK revealed that augmented mortality resulted from delayed treatment for cancer in four weeks. Another inquiry in the UK conducted by Maringe and colleagues (24) indicated that the COVID-19 pandemic leveraged the number of deaths within five years after diagnosis by 4.8–16.6% for esophageal, lung, colorectal, and breast cancer. According to Concepcion et al.’s (25) study, between 2019 and 2020 in the U.S., cancer screening for breast cancer diminished by 16.01% and for colorectal cancer by 24.98%. These reduced cancer screenings might contribute to a rise by 2.89% for breast cancer and a rise by 19.72% for colorectal cancer as well as a rise in the number of breast and colorectal cancer deaths between 2019 to 2021 in the U.S (25).

## What Can be Done to Counteract the Impacts

To counteract the impacts that reduced cancer screening and diagnosis may cause, the following could be implemented. First, while the central government publicly manage and fund population-based cancer screenings in many developed countries such as US, UK, Australia, and New Zealand (26–28), this may not be observed in many other countries, in which affordability for cancer screenings would diminish not only during the pandemic but also during the new normal due to reduced income among the population (28). Therefore, in such countries, the central government should not solely be the key cancer screening organizer but likewise play a central role in mobilizing resources necessary for effective planning and implementation of cancer screening programs (29). The central government should demonstrate some commitment, especially financial commitment to cancer control strategy and cancer screening programs, such as by providing some subsidy for expenses for cancer screenings as well as for subsequent treatments so as to enhance cancer screening participation rates. For instance, in South Korea, cancer patients who have partaken in the national cancer screening program can receive medical payment support from a government program (30). The central government should also mobilize financial resources through public-private partnerships and collaboration with NGO partners (29). The central government should more strongly collaborate with health insurance partners to enhance the coverage for cancer screenings and treatments especially in countries such as Zimbabwe with low health insurance coverage (29).

Moreover, the central government should provide funding and strategic guidance to state or provincial governments and render these governments accountable for the implementation of cancer screening programs (31). Some state governments such as Tamil Nadu in India demonstrate a strong commitment to cancer screenings by ensuring programmatic leadership, mobilizing funds, and building infrastructure and human resources for cancer screening programs (31). In some countries such as South Africa, cancer screening programs (e.g., Pap smear testing) primarily focus on urban settlements, and thus should be decentralized to enhance the effectiveness of cancer screening programs across the country (32).

Policy makers, governments, and healthcare systems should also understand cancer screenings in different areas with different levels of resources. Residents in high-resource areas have concern about how to access services for cancer screenings, while priority for residents in resource-constrained settings entails enhancing their awareness of benefits of cancer screenings. Hence, state or provincial governments should address these diverse needs in implementing cancer screening programs in their states or provinces (33).

Second, resumption of cancer screening should be activated particularly after a lockdown. This can be done through reassuring people of the low likelihood of coronavirus inflection at diagnostic centers. These centers should assure people of preventive health protocols during the performance of cancer screening procedures (34) comprising waiting area rearrangement, personal protective devices, COVID-19 rapid tests, and sanitizers (5).

Third, in addition to encouraging people to visit diagnostic centers through alleviating their fear of coronavirus infection, participation in cancer screening during the pandemic should be enhanced *via* mass media campaigns. Media campaigns have been reported to be cost effective and demonstrate positive long-term health effects (35, 36). Restoring cancer screening participation rates to levels prior to the pandemic should be the objective of media campaigns (20). To do so, media campaigns on television or radio should increase public awareness of cancer risks, benefits of cancer screening, and timely referral benefits particularly in case of suspicious symptoms emerging, as well as prompt those who have postponed or called off appointments due to phobia for coronavirus infection (34).

Fourth, when lockdowns are eased or the pandemic turns to a new normal, demands for catch-up screening may surpass the supply capacity of diagnostic centers, which may lead to long waiting times (20). Hence, the capacity of diagnostic centers should be enhanced by relocating staff with screening expertise to the place of cancer screening work as well as adding to this workforce at the new normal of the pandemic or post-pandemic (34).

Fifth, by virtue of limited resources and higher potential of screening results requiring precancerous or cancerous lesion diagnosis during the pandemic, prioritization for cancer screening should be implemented for high-risk people (3). For instance, mammography screening operations should be prioritized to high-risk women on the basis of family history, history of menstruation, pregnancy, dense breast tissue, or prior breast tumor or cancer, as well as carrying BRCA1 and BRCA2 as high-risk genes of breast cancer (5). Priority for lung cancer screening through low-dose computed tomography (LDCT) and pulmonary nodule stratification should be given to 55-74 year-old people with a smoking history of at least 30 pack-years (7, 37).

Sixth, to address potential shortage of resources, including human resources, for cancer screening during the pandemic as well as prevent COVID-19 spread, different screening modalities are encouraged (4). Telehealth services can serve as an effective option. A positive link has been observed between patients’ telehealth use and cancer screening participation (4). Medical consultation and cancer screening rescheduling can be implemented through telehealth appointments (4, 38). Accordingly, telehealth services should be established and strengthened to help patients with no ability to access diagnostic centers and mitigate face-to-face visits (34). Telehealth services can also incorporate cancer care services following cancer diagnosis such as symptom management, remote chemotherapy supervision, and palliative care (6). As for healthcare staff who undergo self-isolation because of contacting a coronavirus case, telehealth can help them continue provide video or telephone consultations for cancer screening and diagnosis, follow-ups, as well as attend multidisciplinary team meeting for cancer diagnosis and treatment (6).

Furthermore, telehealth services can be integrated with other screening modalities. For instance, some home-based non-invasive screening test kits, such as DNA testing or fecal immunochemical test, can be consulted through telehealth platform (34, 39) and distributed at the door (40). Patient samples can be delivered to laboratories through postal-based system (20) or transport services (41). Screening test results and diagnostic follow-ups can be performed *via* telehealth services again (42).

Last, regardless of benefits of telehealth services for cancer screening, accessibility is still needed for screening participation for some cancer types such as breast cancer (43). Community residents’ accessibility to mammography can be enhanced through outreach programs (11). A flexible outreach system should be built to sustain screening rates for breast cancer in compensation for restriction for cancer screenings in diagnostic centers (11).

## Conclusion

The COVID-19 pandemic has induced disruptions in various services including health services. Particularly, cancer screening and diagnostic services have been substantially dropped in number during the pandemic. Reduction in the number of screenings for lung, prostate, cervical, breast, and bowel cancer has been observed in several countries such as the U.S., the UK, Canada, Australia, and the Netherlands. The worst hit were screening procedures such as colonoscopy and mammography.

Reduced cancer screenings and diagnoses due to the pandemic could exert impacts in the long run comprising increased demands for cancer screenings and diagnoses after pandemic restrictions, enhanced demands for resources in response to such increased demands for cancer screenings, and poor prognosis for cancer patients due to migration to later stages with poor responsiveness to therapies and higher likelihood of mortality. Many measures can be adopted to counteract these impacts after pandemic restrictions, at a new normal of pandemic, or after the pandemic. They may include adopting strong preventive health protocols to alleviate people’s fear of COVID-19 infection at diagnostic centers, encouraging cancer screening participation through media campaigns, enhancing resources including human resources and laboratory capacity for increased screening demands, providing cancer screening priority for high-risk people, encouraging different cancer screening modalities especially telehealth consultations, and adopting flexible outreach system.

## Author Contributions

TL contributed to conception and design of the study, organized the database, performed the analysis, wrote sections of the manuscript, contributed to manuscript revision, read, and approved the submitted version.

## Conflict of Interest

The author declares that the research was conducted in the absence of any commercial or financial relationships that could be construed as a potential conflict of interest.

## Publisher’s Note

All claims expressed in this article are solely those of the authors and do not necessarily represent those of their affiliated organizations, or those of the publisher, the editors and the reviewers. Any product that may be evaluated in this article, or claim that may be made by its manufacturer, is not guaranteed or endorsed by the publisher.
